# Nanobubble-Saturated Water in Soil–Plant Systems: Root-Zone Oxygenation, Chemical Responses, and Structural Stability

**DOI:** 10.3390/nano16150906

**Published:** 2026-07-24

**Authors:** Yeganeh Arablousabet, Arvydas Povilaitis

**Affiliations:** Department of Water Engineering, Vytautas Magnus University, K. Donelaičio g. 58, 44248 Kaunas, Lithuania; yeganeh.arablousabet@vdu.lt

**Keywords:** nanobubble-saturated water, root-zone oxygenation, soil–plant interactions

## Abstract

Nanobubble-saturated water (NBSW) has gained prominence in agricultural research due to its ability to affect root-zone oxygenation and soil–plant interactions. This review aimed to analyze the available research on the impacts of NBSW on soil moisture storage, percolation, electrical conductivity (EC), microbial activity, and soil structural dynamics across different gas types and soil textures. Therefore, a systematic literature search was carried out, and the retrieved publications were synthesized with keyword co-occurrence and bibliometric analysis. This review fills the gap in the literature by integrating findings on root-zone oxygenation, nutrient dynamics, and soil structural responses into a single framework. Research showed that NBSW may influence water partitioning by increasing evaporation and lowering percolation, leading to different vertical moisture gradients. However, the effects could be impacted by the soil texture, as finer textures retained more moisture, while coarser soils increased oxygen penetration and microbial stimulation. Furthermore, increased oxygen availability under NBSW further changed microbial community activity, which could impact soil CO_2_ emissions and nitrogen and phosphorus cycling. While repeated NBSW application may gradually increase soil compaction, particularly in finer-textured soils, overall, NBSW demonstrated versatile watering modifications that might influence soil physical, chemical, and biological processes. However, further research is needed to determine the appropriate gas types and application strategies for different soils under real field-scale agricultural conditions.

## 1. Introduction

Water shortage is a major concern in many regions of the world [1]. Improved water management can reduce the impact of water shortages and partially fulfill the water demand for food production [2]. Due to the limitations of traditional watering systems in terms of water-use efficiency and soil health, innovative technologies have gained a lot of attention [3]. Irrigated agriculture plays a crucial role in global food production, as it accounts for only 20% of the world’s agricultural land yet produces 40% of the world’s food [4]. By 2050, global food demand is anticipated to increase by 70%, while agricultural water use may increase by 19% [5]. Conventional irrigation, which remains dominant worldwide, yields water consumption efficiency of just 50–60%, resulting in significant losses due to runoff, deep percolation, evaporation, and waterlogging [6]. Subsurface drip irrigation is a more effective option, with efficiencies close to 100% and the ability to practically double the irrigated area using the same water allocation [7]. Subsurface emitters, despite their benefits, force oxygen out of the soil pore space by creating a constant wetting front [8,9]. Previous research has examined aeration behavior at the emitter and has reported that aerated water generates a gas–liquid two-phase flow, with a bubbly flow being identified as the predominant regime within labyrinth channels [10]. Bubble movement is governed by inlet pressure and channel geometry, and MNBs reduce drag by changing the local viscosity and density of the flow, which has been linked to increased dissolved oxygen in the emitter effluent and more uniform discharge than venturi-based aeration [10]. This suggests that the gas transported to the root zone is determined by emitter-level hydraulic conditions rather than the aeration technique alone [10,11]. Soil oxygen levels gradually decline as a result of this, disrupting the equilibrium between oxygen use and replenishment [12]. In these circumstances, photosynthesis decreases, root respiration slows, and leaf growth is limited [13]. Plant roots require a steady supply of oxygen to sustain aerobic respiration and the metabolic processes that drive nutrients and water intake [14]. When oxygen levels fall below necessary levels, adenosine triphosphate (ATP) generation declines considerably, ion transport fails, and root growth effectively ceases [14]. Low-oxygen conditions in the rhizosphere caused by compaction, salinity, sodicity, and irrigation have long been recognized as significant obstacles toward reaching yield potential [15,16,17]. One study reported that oxygation increases the soil oxygen content by 2.4–32.6% and boosts microbial metabolism and aerobic root respiration [18]. Yield has been shown to increase by 10–33% in tomato, watermelon, corn, and cotton [19,20,21,22]. The word “oxygation” refers to the aeration of water used for irrigation, using venturi injectors, air compressors, or peroxide addition [23]. Previous research found that air injection and hydrogen peroxide increased cotton lint yield by 28% and 14%, respectively, compared to non-aerated controls. Additionally, water-use efficiency increased from 2.15 to 3.65 g/L [9]. Similar responses have been reported in radish, where air injection increased yield by 9.87% over conventional drip irrigation [23], whereas oxygation of cotton in vertosols corresponded to increases of 17% in total root mass per plant, 53% in root length density, and 26% in taproot mass [22]. These results demonstrate that under irrigated conditions, a root-zone oxygen supply limits yield. However, because the supplied gas volume cannot be precisely controlled and the air provided as macrobubbles tends to move away from the root zone, the same body of work indicated that this restriction drives NB technology [23,24,25]. The primary limitation of conventional oxygation systems employing venturi injectors, air pumps, or hydrogen peroxide is that they create large-sized bubbles that rise fast and rise above the soil before oxygen can effectively reach the root zone [24,25]. Nanobubble (NB) technology addresses this concern. NBs are tiny gas bubbles less than 1 μm in diameter. They have a large specific surface area, low rising velocity, high gas solubility, and high mass transfer rates, which allow them to stay in water for a longer period than macrobubbles [26,27]. NBs enhance gas–liquid transfer efficiency in various applications, such as water treatment, agriculture, food processing, and healthcare, according to research conducted over the past 20 years [28]. Their negatively charged surface throughout a wide pH range enhances their suspension stability [29]. The oxygen transfer rate in NBs was determined to be 125 times that of macrobubble treatment [28]. According to studies, NBSW increases crop production and water-use efficiency by 16.9% in tomato and 22.1% in cucumber, but with no increase when using water or fertilizer input, contributing to enhanced soil oxygen content [30]. In addition to yield, as confirmed by structural equation modeling, NBSW alters the functional capacity of bacterial communities in the rhizosphere, improves soil fertility indicators, and increases crop productivity using both direct and indirect pathways [31,32]. In controlled agricultural environments, where circular water systems frequently produce hypoxic root zones, low dissolved oxygen levels have been linked to decreased plant growth, decreased microbial activity, the accumulation of harmful metabolites, and an increased susceptibility to root diseases [33]. Oxygen nanobubbles (ONBs), which have a high oxygen-carrying capacity, have been shown to maintain high dissolved oxygen concentrations, support beneficial microbial populations, and strengthen plant resilience without the need for continuous mechanical aeration [33]. Air and oxygen NBSWs are the most commonly used types in agricultural watering due to their accessibility and compatibility with current watering systems.

The effects of NBs on soil moisture, nutrient dynamics, and plant performance vary based on gas type, soil texture, and exposure duration [34,35,36]. Different types of NBs, including air, N_2_, O_2_, H_2_, Ar, and CO_2_, show the potential of a varied gas supply, which emphasizes their adaptability and wide range of use in many industrial and agricultural contexts [34]. Zeta potential, which is directly connected to the solubility of gases and diffusion properties of the resulting bubbles, has been demonstrated to be strongly influenced by the kind of gas utilized in NB formation [34]. In contrast to oxygen NBs, which exhibit a higher initial oxygen diffusion potential but a shorter bubble lifespan due to rapid collapse, air NBs have shown higher stability and longer dissolved oxygen retention [35]. ONBs tend to speed up organic matter decomposition under high evaporative demand, whereas air NBs more frequently support short-term moisture retention, especially in coarser-textured soils [35,36]. Since the same type of gas can produce different outcomes depending on soil texture, organic matter content is also an important factor that influences plant responses to NB watering [36,37].

The majority of current research focuses on crop yield, water-use efficiency, or soil fertility without thoroughly investigating the physiological links among root-zone oxygenation, nutrient availability, and water retention in the root zone and plant responses such as stomatal conductance, chlorophyll content, and nutrient uptake [4,31,33]. Although root-zone oxygenation is typically considered the most important aspect impacting plant performance with NB watering, NBs’ capacity to sustain nutrient availability for absorption and to retain water in the active root zone is also essential to plant growth outcomes. However, these characteristics have rarely been studied together within a coherent mechanistic framework [18,35,36]. Addressing this gap is crucial because oxygenation, nutrient availability, and root-zone water retention are the primary processes through which NBSW is expected to affect plant growth and production.

The objectives of this review were to (1) analyze how NBSW affects root-zone oxygenation and soil moisture storage in various soil textures and types of NB gases; (2) synthesize the existing data regarding the impact of NB watering on nutrient availability, leaching patterns, electrical conductivity (EC), and interactions among soil microorganisms and physicochemical properties; and (3) assess the temporal and structural characteristics of NB watering and to examine its temporal and long-term impacts on soil compaction and water balance.

## 2. Materials and Methods

To address the objectives, the current review summarizes and integrates the existing literature on soil-level changes caused by NBSW. In order to formulate findings on these mechanisms, a thorough evaluation of the research literature on NBSW and its impacts on soil was carried out. The data analysis contributes to the literature by providing the following insights:

- The significance of root-zone oxygenation in soil moisture storage and water retention across various soil textures and NB gas types;
- EC, leaching patterns, and nutrient availability in response to NB watering;
- NBSW application-related soil microbial and physicochemical interactions;
- Temporal and structural soil responses to NBSW during both transient and long-term watering episodes.

**Figure 1:**
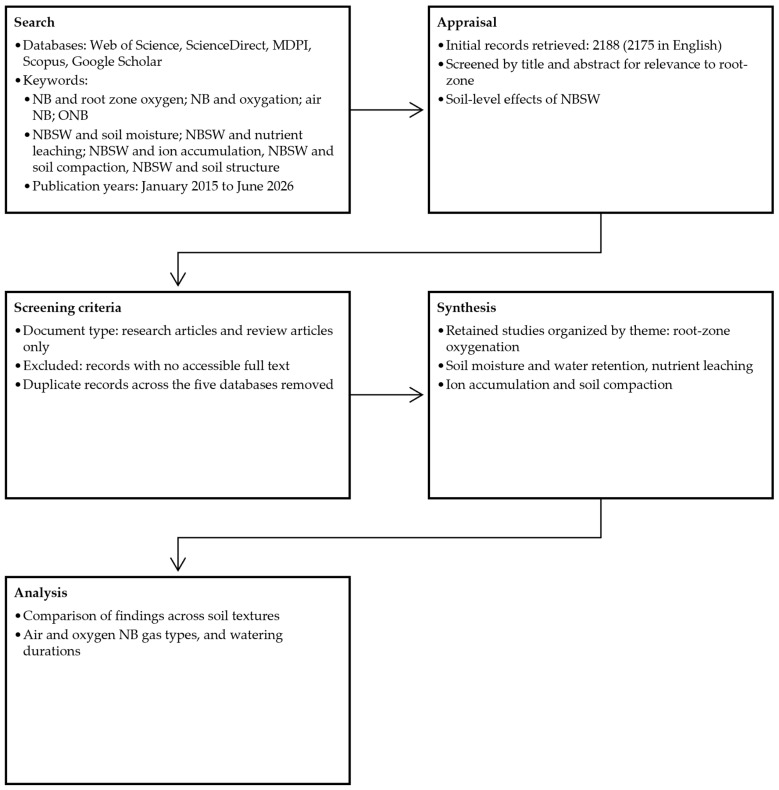
The process of the systematic literature review.

This review highlights the current state of knowledge on soil-level responses to NBSW using the SALSA approach, which includes a scope search, appraisal, analysis, review, and refinement of data. According to the scope of this review, keywords were classified into two categories. The first category focused directly on root-zone oxygen delivery (NB and root zone oxygen, NB and oxygation, air NB, and ONB). The second category focused on the soil-level effects of this oxygenation (NBSW and soil moisture storage, NBSW and nutrient leaching, NBSW and ion accumulation, NBSW and soil compaction, NBSW and soil structure). The review was limited to research and review publications published between January 2015 and June 2026, and were retrieved from Web of Science, ScienceDirect, MDPI, and Scopus; of the 2188 generated results, 2175 were in English. The search process is shown in Figure 1.

**Figure 2:**
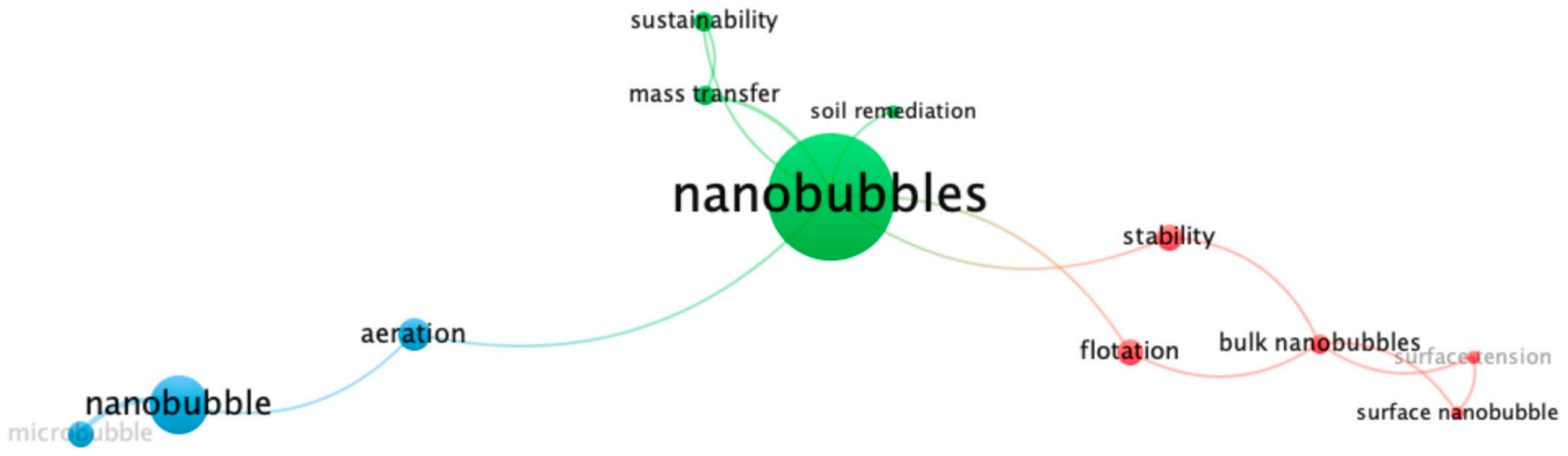
A network visualization map of the primary keyword “nanobubbles” based on the WoS database.

The titles and abstracts of the 2175 English-language records were filtered for direct relevance to root-zone oxygenation and the soil-level effects of NBSW, whereas records focusing mostly on water treatment, industrial, or unrelated agricultural uses were excluded. Peer-reviewed journal publications and review papers on NBSW applications in soil–plant or agricultural systems were included; conference papers, non-peer-reviewed sources, and research with no demonstrable soil-related effects were eliminated. This filtering technique provided 72 publications, which were selected for full-text examination and keyword analysis. VOSviewer (version 1.6.20) was used to conduct a keyword co-occurrence analysis on the keywords derived from the 72 publications obtained through the structured literature search. After applying a minimum occurrence threshold and deleting terms irrelevant to the topic of this research, 13 keywords fulfilled the criteria for inclusion and were divided into three separate clusters (Figure 2). The green cluster focuses on the phrase “nanobubbles”, which is the primary linked keyword appearing in the cluster. This cluster comprises the terms “mass transfer”, “sustainability”, and “soil remediation”, which indicate NBs’ critical role in supporting gas transfer processes and their significance to sustainable soil management techniques. The association of “nanobubbles” and “soil remediation” emphasizes the clear connection between NB application and soil-level treatments, which forms a conceptual connection to root-zone and agricultural applications. The blue cluster, which includes “nanobubble”, “microbubble”, and “aeration”, highlights the fundamental terminology that distinguishes NBs from microbubbles, while highlighting aeration as a main functional use. The connection to “aeration” between this cluster and the green “nanobubbles” cluster suggests that oxygen supply and gas transfer continue to be a unifying subject in both fundamental and practical NB research. The red cluster contains the terms “stability”, “flotation”, “bulk nanobubbles”, “surface nanobubbles”, and “surface tension”, which reflect the physicochemical parameters that regulate NB behavior in liquid media. The prominence of “aeration” and “stability” in this keyword network aligns precisely with the structure of this review, which starts with an examination of the mechanistic basis of NB stability and oxygen delivery before moving on to the soil moisture, nutrient, and structural consequences in subsequent sections. Black labels represent terms with a higher co-occurrence frequency, whereas grey labels indicate keywords with a lower co-occurrence frequency within the network. The distance between nodes shows the degree of association, and the closer proximity suggests more co-occurrence relationships.

**Figure 3:**
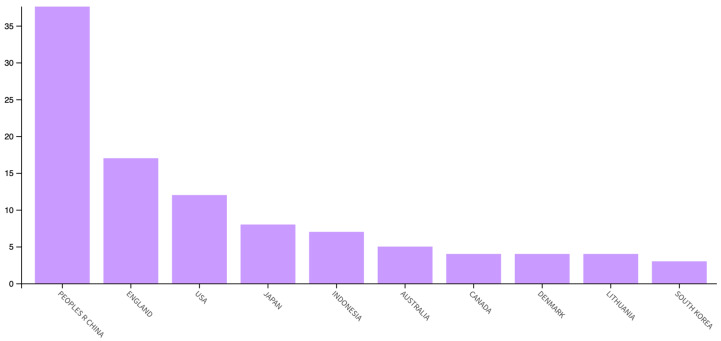
Analysis of countries with the highest number of publications in 2015–2026 based on the WoS database (Note: country totals exceed the number of included publications (*n* = 72) since publications with authors from multiple countries are counted once per contributing country).

**Figure 4:**
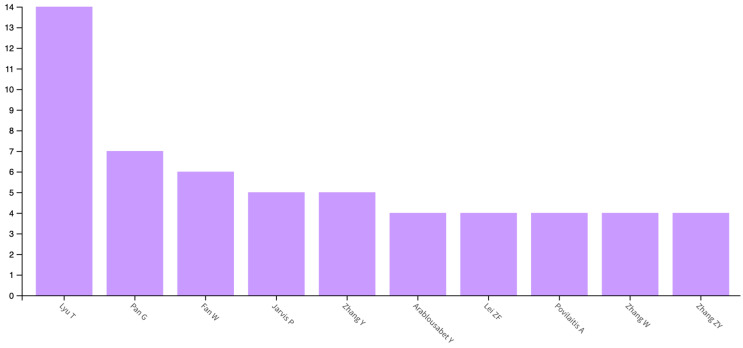
Analysis of authors with the highest number of publications in 2015–2026 based on the WoS database.

**Figure 5:**
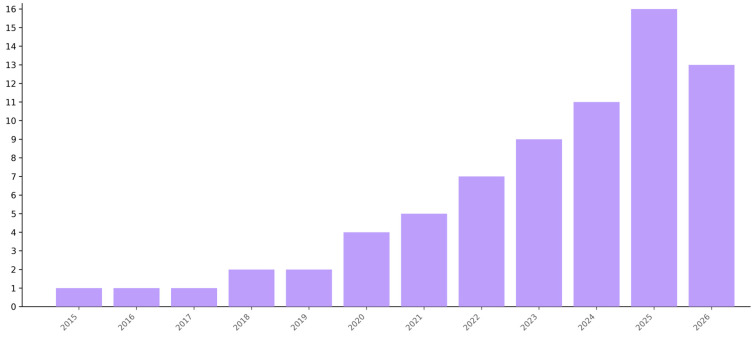
Analysis of the number of publications per year (2015–2026) based on the WoS database.

The country-level analysis of the relevant keywords mentioned above illustrated that China had the highest number of publications on this topic (38), followed by England (17), the United States (12), Japan (8), Indonesia (7), and Australia (5). Canada, Denmark, and Lithuania each recorded 4 publications, while South Korea had 3 (Figure 3). Furthermore, according to the author analysis, Lyu, T. (14) had the highest number of publications, followed by Pan, G. (7); Fan, W. (6); Jarvis, P. (5); and Zhang, Y. (5). Povilaitis, A.; Arablousabet, Y.; Lei, Z.F.; Zhang, W.; and Zhang, Z.Y. each contributed 4 publications (Figure 4). Figure 5 further presents the number of articles published each year from January 2015 to June 2026 on this topic. The upward trend illustrates the fast-growing research interest in NBs and its soil–plant applications, which confirms that the field has grown in recent years. The PRISMA 2020 checklist is provided in the Supplementary Materials.

## 3. Root-Zone Oxygenation and Soil Moisture Storage

The persistence of a bubble is typically assessed by how much oxygen the NB can deliver to the root zone and diffuse into the surrounding soil solution [38,39]. The evaluation of dissolved oxygen (DO) decay in air and ONB water revealed a significant difference in stability between the two gas types. The previous studies showed that ONBs in water lost 23% of their DO within the first half hour after generation, while air NBs lost 6%; this gap increased further, as ONBs lost 3.2 times more DO than air NBs between 5 and 60 h, which was the time it took for the DO to reach the same level [35]. The observed increase in soil oxygen concentration indicates improved oxygen transfer, retention, and diffusion facilitated by the presence of the bubble in the solution [40]. Electrostatic effects at the gas–liquid interface have been connected to the structural foundation of NB stability. It has been reported that the negatively charged ions accumulate on the surface of the bubble and build up a protective film, which slows gas diffusion outward. Meanwhile, the electrostatic repulsion between nearby bubbles prevents them from integrating into larger and less stable forms [38]. After about 60–70 h, both air and oxygen NBSW converge with conventional water [35]. However, the oxygen decay accelerates further once the NB moves into the soil matrix, since the minerals and organic surfaces quickly destabilize it [35]. The NB gas composition is also considered a contributing factor to the stability gap. For instance, in contrast to ONB, which more quickly diffuses oxygen content, air NBs primarily comprise nitrogen, which lowers intermolecular bonding in water and contributes to bubble longevity [35]. This idea of slow, surface-mediated release rather than direct gas delivery is supported by the low level of oxygen that a single bubble contains. It has been reported that, while ONBs overall increased the DO level by 14 mg/L, the bubbles themselves contributed about 0.005 mg/L of this increase due to their tiny size and restricted gas volume that each one can physically hold [41]. Further studies at the field level also explained that daily ONB watering supplied about 2% of the oxygen that soil microbes and roots actually consume through respiration [42].

The oxygen that NB carries, before entrapment, varies depending on the soil texture [36]. The findings on different particle sizes of sands revealed that lowering the sand particle size gradually limited NB movement, which resulted in increasing levels of entrapment. Retention was highest in fine sand at 50.8%, decreased to 43.8% in medium sand, and was lowest in coarse sand at 23.5% [43]. Furthermore, clay minerals appear to intensify this restriction, as kaolinite has been shown to attract NBs into subsequently adhering to clay particle surfaces and physically block pore spaces [44]. Environmental factors can further influence this stability balance. For instance, water chemistry changes, temperature, or dissolved substances can affect the surface charge distribution and the stability of NBs [45,46,47]. Additionally, NBs have also been found to restructure flow, adhering to the soil they move through, and increase both permeability and total pore volume after repeated applications [48].

Other research has supported the idea of a steady, distributed oxygen increase rather than a spike in mass supply. NBSW has been shown to increase the oxygen diffusion capacity, respiration rate, and DO saturation during the watering events. It also increases several soil enzyme activities involved in nutrient cycling [49]. Other studies have shown that the soil treated with NBSW resulted in a reduction in microbial biomass; however, the microbial diversity remained unaffected [49]. Moreover, venturi-based aeration has been associated with increased soil permeability and a more even distribution of water across the profile, which allows water to penetrate deeper into the root zone than conventional watering [50]. However, more recent studies have suggested there are advantages of NB generators over venturi injectors: MNB aeration produces more dissolved oxygen in the emitter effluent, more uniform discharge, and a longer delay before emitter clogging [10]. In comparison to untreated soil, NBSW treatments have resulted in significant increases in root length, surface area, volume, and overall root activity. This response is partially attributed to the activity of auxin, a hormone essential to root development [51]. Particularly, it has been reported that, in moderately salty tomato, root diameter and root volume were significantly enhanced by ONBs with higher DO concentrations compared to lower oxygen levels [52]. Tomato root diameter, volume, and length all increased significantly under a high-versus-low comparison of different NB treatments [53]. Another study found significant increases in tomato root length and volume under NB treatment relative to untreated controls [54]. Increased root surface area, length, and branching are indications of above-ground alterations linked to the improvement in root growth caused by oxygen delivered by NBs. Higher leaf net photosynthetic rates, transpiration rates, stomatal conductance, and increased catalase and peroxidase activity are all correlated with this oxygen supply [55].

The impact of NBSW on oxygen delivery, which contributes to improving soil moisture storage, depends on environmental conditions. It has been shown that switching from air NBs to ONBs under high vapor pressure deficit (VPD) reduced soil moisture by 30.5% in silty clay loam and by 66.1% in sandy loam relative to the soil treated with conventional water over the same period [35]. Since the additional oxygen from ONBs is considered to increase microbial respiration and speed up the breakdown of organic matter, weakening soil aggregates and increasing the exposure of soil to evaporation, this loss has been linked to a biological rather than a physical cause [35]. However, when the condition changed, this trend reversed, as sandy loam under air NBs contained 13.3% more moisture than the conventional control when the VPD decreased, and air NBs were reapplied; silty clay loam showed a less significant but comparable reversal [35]. It has been revealed that NBSW did not show a statistically significant change in soil water-holding capacity (*p* > 0.05) compared with conventional water. Long-term soil moisture storage was primarily determined by soil texture and cumulative water input, rather than NB watering [36]. However, NBSW affected soil water partitioning through evaporation and percolation. It led to an increase in the cumulative evaporation in silty clay loam and sandy loam soils, but the leachate volume decreased substantially during the same time period. Furthermore, this similar redistribution was seen in the vertical profile, in which the topsoil layer contained more moisture than the deeper layer significantly more frequently under NBSW than under conventional watering in silty clay loam. This occurred in 37.2% of measurements under NBSW vs. under conventional watering [36]. In sandy loam, the texture-dependent pattern changed. Conventional watering resulted in a higher frequency of topsoil moisture exceeding deeper-layer moisture than NBSW, occurring in 54.9% of conventional water measurements versus 34.9% of NBSW measurements [36]. NBSW has also been reported to show much higher variation than conventional water, which generated a more stable vertical moisture distribution across soil layers in sandy loam [36].

## 4. Nutrient Leaching, EC, and Microbial Responses

According to the studies, NBSW did not show a significant difference in nutrient leaching (such as nitrate (NO_3_^−^), phosphate (PO_4_^3−^), sulfate (SO_4_^2−^), calcium (Ca^2+^), iron (Fe^3+^), and various heavy metals) compared to conventional water-treated soil [35]. However, depending on the gas type, air NBSW was consistently associated with lower leachate concentrations of NO_3_^−^, PO_4_^3−^, SO_4_^2−^, total dissolved solids, Ca^2+^, Fe^3+^, and heavy metals, such as Cr and Cu, than conventional watering during most observation periods [35]. Total dissolved solids in the input water have been observed to be 6–15% lower with NBSW compared to conventional water [35]. Meanwhile, ONB watering resulted in a less uniform pattern that was dependent on soil texture. It has been documented that ONB watering increased the leaching of PO_4_^3−^, Mn, Cu, and chemical oxygen demand (COD) compared with conventional water in silty clay loam soil. However, it reduced the leaching of NO_3_^−^, SO_4_^2−^, Fe^3+^, Cr, and Ca^2+^ [35]. While sandy loam soil treated with NBSW contributed to the restricted downward water movement and, as a result, negligible leaching of the nutrients, which retained water and dissolved substances mostly restricted to the topsoil layer [35]. It has also been reported that in sandy loam, the collected leachate volume was 0.1 L under NBSW compared to 4.9 L under conventional watering over the same period, and leaching losses with NBSW contributed to 0.2% of the applied water compared with 8.5% under conventional watering [35]. However, the primary reason was explained to be due to the compaction induced by ONB watering, which resulted in the restricted downward movement of water and, ultimately, in the absence of nutrients [35,38]. Additionally, different responses have been observed between air NB and ONB treatments from a microbial activity perspective, as ONB boosts organic matter decomposition and the subsequent release of certain ions while also increasing the binding of others, such as iron and chromium, to compost-derived organic matter, which would explain why some nutrients showed increased leaching [35]. Nutrient ions, such as K^+^, Na^+^, Ca^2+^, Mg^2+^, and NH_4_^+^, were reported to be drawn to the negatively charged surface of the NB; this mechanism has been suggested to explain why such ions may become more available for root uptake rather than being lost to drainage [38]. Different gas types seem to mobilize nutrients in different ways. Nitrogen-filled NBs exhibit a particularly strong correlation with nitrogen availability, which is partially explained by direct nitrogen intake and partially by the higher surface charge of nitrogen NBs in comparison to other gas types [38]. Carbon dioxide NBs (CNBs) have been linked to a specific mechanism, increasing the release of soil cations, including Ca^2+^ and Mg^2+^, through moderate soil acidification rather than surface-charge attraction [38]. Furthermore, increased DO concentration due to NB watering has been related to a change in microbial nutrient cycling pathways overall, including greater rates of nitrogen mineralization, which makes organic nitrogen more accessible for plant use [56]. A similar pattern has been reported for phosphorus, where increases in oxygen availability due to NBs have been linked to increased phosphatase activity, which is associated with higher phosphorus accessibility for plant absorption [57].

EC has been reported to be more soil dependent. The studies showed that in silty clay loam, EC reduced under air NBs compared to conventional water-treated soil during the warmer periods. However, during the cooler periods, EC increased, a shift that was attributed to improved infiltration and moisture storage under air NBs [35]. In sandy loam, EC under NBSW was 24% lower under ONB and 4% lower under conventional watering during the warmer period. However, when air NBs were reapplied under colder conditions, EC increased by 16%, and it stayed 14% higher during the succeeding ONB period [35]. The studies on sandy loam soil revealed the higher decomposition of organic matter, which could be attributed to the higher EC under air and oxygen NBSW-treated soil in comparison with conventional water treatment [35]. This texture-dependent EC response is attributed to structural soil changes caused by ONB, in which decreased infiltration and increased compaction retain water and dissolved ions near the surface, thereby concentrating solutes in the topsoil and increasing EC even in the absence of high overall moisture content [35,38]. Further studies analyzed the EC under saline rather than non-saline conditions. In saline–alkali soil, NBSW treatment, in combination with a microbial component, lowered EC over time, which suggests a decrease in water-soluble salts in the topsoil [58]. The same treatment reduced soil pH by 5.8%, compared to a 5% reduction with NBSW application alone, which was attributed to microorganisms’ ability to neutralize alkaline soil conditions [58]. In cotton fields treated with NBs, a similar pattern of decreased surface salinity has been observed; topsoil salinity decreased across a range of initial salinity levels while salt concentrations in deeper layers increased correspondingly. This suggests that the salts were actively transported downward through the profile rather than just evaporating near the surface [44].

Additionally, it has been demonstrated that soil microbial activity responds to oxygation caused by NBs in ways that impact nutrient cycling instead of immediate leaching. Enhanced nitrogen mineralization and enhanced activity of nitrogen-assimilation enzymes, such as nitrate reductase and glutamine synthetase, have been associated with improved DO conditions related to NBSW [59]. At the same time, NBSW has been linked to a lower transient risk of NO_3_^−^ accumulation, which is attributed to the inhibition of nitrite-oxidizing bacteria, thereby restricting NH_4_^+^ conversion to NO_3_^−^ [60]. Soil CO_2_ emissions, which are used to determine microbial respiration, have been recorded as higher under NBSW than under conventional water, with the increase being more prominent in sandy loam than silty clay loam soil, which could prove the idea that the coarser soil structure allowed oxygen from the NBs to reach deeper and promote microbial activity more efficiently than in the finer-textured silty clay loam [35]. Sandy loam showed a more significant rise in EC over time under NBSW compared to conventional water, whereas silty clay loam’s EC level under NBSW showed a clear upward trend throughout the monitoring period [36]. This persistent difference in EC was interpreted as evidence of greater dissolved ion retention within the soil profile under NBSW, which is consistent with the lower leaching volumes observed under NB treatment compared to conventional watering over the long-term period [36]. Recent research has expanded similar findings to ozone NBs, which are increasingly used in aerated drip irrigation. Ozonation of the watering process raised the level of dissolved oxygen from 4–5 to 9–10 mg L^−1^, and was correlated with higher available nitrogen and potassium, increased urease activity, and greater bacterial community richness in the maize rhizosphere. Structural equation modeling indicated that these microbial changes were primarily mediated by improved soil fertility [61]. At the gene level, ozonated water enriched nitrogen-fixing species and raised the quantity of the nitrogen fixation gene nifK [62]. Denitrification genes have been shown to respond differently depending on their position in the pathway, as the gene norB, which encodes a later step, was highest under non-aerated treatment and did not increase significantly with ozonation, indicating partial rather than complete denitrification and thus a lower potential for gaseous nitrogen loss [62]. These gene-level findings are consistent with the nitrogen-retention responses described for air and oxygen NBs, and they point to microbial mechanisms by which enhanced oxygen conditions can restrict nitrogen loss in the root zone.

## 5. Soil Compaction and Long-Term Soil Water Balance

Repeated application of NBSW may cause soil compaction if not combined with mechanical loosening techniques, which is more apparent in silty clay loam than in sandy loam soil [36], while also increasing microbial activity that contributes to this compaction. However, air NBs demonstrated potential to decrease compaction in the short term [35]. Moreover, the soil texture has a significant role in compaction responses as well. For instance, the sandy loam soil has been recorded to have significantly lower compaction than silty clay loam soil [35]. The average soil moisture-weighted compaction under NBSW has been reported to reach 11.36 kg/m^2^ in silty clay loam compared with 7.56 kg/m^2^ under conventional watering. However, in sandy loam, the corresponding values were 6.32 and 5.30 kg/m^2^ [36]. Nevertheless, the average observed compaction under NBSW treatment was higher than that under conventional watering treatment, which was associated with a moisture-weighted increase in compaction relative to the conventional treatment [35,38]. This compaction seemed to impede the effectiveness with which water could move downward through the soil profile, as the consistently higher moisture content recorded in the deeper layer indicated that water was remaining near the surface rather than infiltrating. However, this pattern was more visible in silty clay loam than in sandy loam [35]. Since the water was retained nearer to the surface instead of penetrating deeper into the profile, therefore the topsoil layer was more exposed to evaporation and faster drying [35]. The effectiveness of NBs to reduce root-zone hypoxia may be limited in highly compacted soils when gas transport movements are impeded [34]. According to the reports, NBSW increases the soil compaction in soils that are rich in organic matter, specifically under ONB treatment. ONB treatments showed an increase in aerobic microbial activity, while air NBs, in contrast, may temporarily reduce the compaction and improve the water pathway, which is attributed to a more balanced gas composition [35]. This microbially induced structural modification is particularly connected with the disintegration of soil macroaggregates following extensive aerobic decomposition of organic matter in high-oxygen conditions [63]. The type of gas and the duration of application affect the relationship between NBSW and soil aggregate stability. ONB applications have been linked to a structural rearrangement of the soil matrix, where aggregate cohesion decreases while porosity and pore connectivity increase, driven by microbially mediated organic carbon decomposition under high-oxygen conditions [48]. Air NBs, on the other hand, have been shown to temporarily reduce compaction and improve soil-water interactions due to their more balanced gas composition, which limits the aerobic decomposition cycle associated with ONBs [35]. The negatively charged and less reactive properties of air NBs, which may improve short-term interactions between soil and water, are consistent with the general physical concept of NB stability, in which a charged film at the gas–liquid interface prevents outward gas diffusion and helps prevent individual bubbles from coalescing into larger, less stable forms [38]. Other studies have demonstrated that ONBs significantly reorganize the network, altering the pore-size distribution toward finer micropores and increasing micropore porosity many times. In contrast, air NBs had only minimal, non-significant effects [64]. This finer-pore development has been attributed to a combination of physical and physicochemical processes, including hydraulic disturbance as bubbles rise and collapse, as well as the influence of the charged bubble surface on interparticle forces, which are thought to disperse aggregates and promote micropore formation [64]. When combined with the microbially mediated decomposition of macroaggregates observed under persistent high-oxygen conditions, Ref. [63] has suggested that ONB treatment likely drives structural turnover rather than consolidation. Moreover, a higher density of tiny pores does not imply a better-connected system, as increases in pore number and specific surface area have been followed by decreased pore connectivity, indicating a shift toward more isolated pore spaces rather than continuous transmission pathways [64]. Since such evidence is primarily from flooded, waterlogged systems and characterizes pore architecture rather than typical aggregate-stability indices, it demonstrates that NBSW reshapes soil structure in a gas- and context-dependent manner rather than uniformly improving structural stability.

Since the difference between NBSW and conventional water in cumulative moisture storage has been shown to remain statistically insignificant throughout the entire monitoring period, this long-term structural change in the soil has been reported without any corresponding change in the total amount of water the soil retained overall [36]. Instead, the topsoil layer correspondingly retained more moisture than the deeper layer, and far more frequently under NBSW than under conventional watering in silty clay loam. This suggests that the structural change was reflected in where the water that remained in the soil tended to accumulate [36]. The studies showed that the long-term monitoring of NBSW treatment shifted the water partitioning with higher loss through evaporation and less percolation in both silty clay loam and sandy loam soil [36]. A different pattern of the constrained downward water movement has also been documented under saline field conditions, in which NBSW has been shown to reduce salinity in the topsoil along with increased salinity in deeper layers, which implies that water and dissolved substances were still able to move downward despite surface-level structural changes [65]. Since the degree of restriction seems to vary between soil types, salinity levels, and experimental conditions rather than following a single consistent pattern, the findings may establish a limit on how thoroughly downward water movement is reduced after NBSW treatment. Taken together with the previously mentioned progressive increase in compaction, this change in direction of water loss toward higher evaporation rather than percolation may suggest a structural change in the soil that occurs under repeated long-term NBSW applications. This change has a greater impact on water-loss pathways and root-zone solute dynamics than it does on the total amount of water the soil can store [36]. Future research should further investigate how different NB gas types perform across a broader range of soil textures and timescales, why the NB-induced limitation of downward water and solute movement persists in some soils but not in others, and how NB-driven soil changes affect plant physiology and yield. Research that employs various gas combinations could demonstrate whether gas composition can balance oxygenation, microbial decomposition, and structural change. Future research should investigate how gas concentration, application interval, watering amount, and fertilizer distribution interact across soils and crops to determine when repeated application is helpful and when it risks compaction or limited water movement. One limitation also relates to the scale of the experiment. The majority of the studies discussed here are derived from research conducted in greenhouses, soil columns, and controlled pots. The application of NBs in field conditions differs significantly from controlled environments in several aspects, including soil layering and heterogeneity, groundwater, rainfall, crop rotation, and year-to-year variability. Any of these can reduce, increase, or reverse the observed impacts. Moreover, the current findings, primarily based on pot and controlled experiments, describe processes that occur predominantly in the topsoil layer, and further field-scale research may reveal different patterns. Therefore, results derived mostly from controlled experiments should be considered suggestive, and the application of NBSW in the field should be regarded as provisional until assessed by long-term experiments conducted in different areas.

## 6. Summary and Conclusions

This review confirmed that the soil-level effects of NBSW are highly dependent on gas type, soil texture, and application time, rather than functioning as a single, uniform mechanism across agricultural soils. In soil systems, NBSW has a dual role in controlling water and solute transport. NBSW significantly changed the water partitioning by increasing evaporation and reducing percolation, which leads to distinct vertical moisture gradients. The effects were highly influenced by soil texture. Finer-textured soils retain more moisture, and coarser soils demonstrate increased oxygen penetration and microbial stimulation. The vertical distribution of moisture in the soil profile was also impacted by texture, as in silty clay loam, NBSW increased the frequency of topsoil moisture higher than deeper-layer moisture when compared to conventional water, while in sandy loam, it decreased. Air NBs typically lower nutrient leaching; however, NBs may increase nutrient mobilization depending on the soil structure and organic matter content. Ion-specific responses, such as changes in NO_3_^−^, PO_4_^3−^, and Fe^3+^ behavior, demonstrate the complexities of nutrient redistribution in various NB regimes. EC exhibited a similar soil-dependent pattern, increasing more in sandy loam than in silty clay loam during NBSW treatment. This result was connected with increased organic matter decomposition, which resulted in the release of ions into the soil solution. Furthermore, changes in the microbial community caused by increased oxygen availability have a significant role in controlling nitrogen and phosphorus cycling. This was reflected in increased nitrogen mineralization and increased activity of nitrogen- and phosphorus-related soil enzymes under NBSW, as well as higher soil CO_2_ emissions, which are used to indicate microbial respiration under NBSW than under conventional water in both silty clay loam and sandy loam soil. Repeated NBSW application may affect soil aggregation, compaction, and water movement; however, these effects may vary depending on the type of soil and gas composition. Long-term NBSW application indicated that soil compaction increased gradually over time under both watering treatments. However, the effects were seen to be stronger under NBSW and greater in silty clay loam than in sandy loam soil. Additionally, under specific conditions, air NBs improve soil permeability in the short term, while ONBs are highly associated with increased microbial respiration and aggregate decomposition. Overall, NBSW showed versatile watering modifications that may affect soil’s physical, chemical, and biological processes. It should be highlighted that the majority of the reviewed findings are based on controlled pot experiments that represent the topsoil layer processes, while field-scale studies may reveal different patterns.

## Data Availability

This review article is based on an analysis of the existing literature. No original data or models for this study were generated.

## Supplementary Materials

The following supporting information can be downloaded at: https://www.mdpi.com/article/10.3390/nano16150906/s1, Table S1: PRISMA 2020 checklist for systematic reviews.
