# Supplementary material for: Nanobubble-Saturated Water in Soil–Plant Systems: Root-Zone Oxygenation, Chemical Responses, and Structural Stability
Source: Nanomaterials (Basel). 2026 Jul 24;16(15):906. doi: 10.3390/nano16150906 (PMC13467965; doi:10.3390/nano16150906)
Supplement: Supplementary file 1 [file nanomaterials-16-00906-s001.zip › nanomaterials-4439237-supplementary.pdf]

PRISMA

PRISMA 2020 Checklist

Table S1: PRISMA 2020 checklist for systematic reviews.

Section and Topic	Item #	Checklist item	Location where item is reported
**TITLE**			
Title	1	Title: Nanobubble-Saturated Water in Soil–Plant Systems: Root-Zone Oxygenation, Chemical Responses, and Structural Stability<br>the word “review” has been added at the top of title.	Page 1
**ABSTRACT**			
Abstract	2	Including objectives, method, key results, and conclusion	Page 1
**INTRODUCTION**			
Rationale	3	It describes the global water shortage, limitations of conventional watering treatments, NB technology, NB characteristics, gap in the existing literature for instance root-zone oxygation, nutrients availability, soil water storage, have been rarely studied together within a coherent mechanistic framework.	Page 1&2
Objectives	4	The objectives of this review were to (1) analyze how NBSW affects root-zone oxygenation, and soil moisture storage in various soil textures and types of NB gases; (2) synthesize existing data regarding the impact of NB watering on nutrient availability, leaching patterns, electrical conductivity (EC), and interactions among soil microorganisms and physicochemical properties; and (3) assess the temporal and structural characteristics of NB watering and to examine its temporal and long-term impacts on soil compaction and water balance.	Page 3
**METHODS**			
Eligibility criteria	5	Included peer-reviewed research and review articles from 2015 to 2025 in English language, related to NBSW applications in soil-plant and agricultural systems.<br>The water treatment, industrial NB application, non-peer-reviewed sources, studies which has no soil related effects, were excluded.<br>Studies were grouped into three thematic categories matching the three objectives: (1) oxygenation and moisture, (2) nutrients/EC/microbial, (3) compaction and long-term water balance.	Page 3
Information sources	6	Web of Science, ScienceDirect, MDPI, Scopus. Search limited to publications from 2015 to 2025.	Page 3
Search strategy	7	Two keyword categories were used: (1) root-zone oxygen delivery (NB and root zone oxygen, NB and oxygation, air NB, ONB); (2) soil-level effects — NBSW and soil moisture storage, NBSW and nutrient leaching, NBSW and ion accumulation, NBSW and soil compaction, NBSW and soil structure.<br>Filters: English language, 2015–2025, research and review articles only	Page 3
Selection process	8	2188 records were identified across 4 databases; 13 non-English records were excluded; 2175 English-language records were screened by title and abstract; records focusing on water treatment, industrial, or unrelated uses were excluded; 72 publications were selected for full-text examination. No automation tools were used.	Page 3&4
Data collection process	9	72 full-text publications were reviewed, and data were extracted from 59 through narrative reading and synthesis. VOSviewer was used for keyword co-occurrence analysis of author keywords from the publications. No automation tools were used for data extraction.	Page 3&4
Data items	10a	Root-zone oxygenation, soil moisture storage, water retention, nutrient availability, leaching patterns, electrical conductivity (EC), microbial activity, and soil compaction. All relevant results from each included study were incorporated into the narrative synthesis.	Page 3
	10b	Soil texture (silty clay loam, sandy loam, sandy soils), NB gas type (air, oxygen, CO_2, N_2, and others), application duration (transient vs. long-term), and experimental setting (field, greenhouse, or laboratory). Studies with insufficient soil-related data were excluded rather than assumed.	Page 3
Study risk of bias assessment	11	No formal risk of bias assessment tool was used. This review is a narrative synthesis, and no systematic quality appraisal instrument was used.	
Effect measures	12	Not applicable	

Section and Topic	Item #	Checklist item	Location where item is reported
Synthesis methods	13a	Based on their primary topic and reported outcomes, the studies were assigned to one of three thematic synthesis groups, corresponding to the three review objectives: (1) root-zone oxygenation and soil moisture, (2) nutrient leaching, EC, and microbial responses, (3) soil compaction and long-term water balance.	Page 3&4
	13b	Not applicable	
	13c	Results were described with citations throughout Sections 3–5. Figure 1 shows the PRISMA flow diagram (page 4). Figure 2 shows a VOSviewer keyword co-occurrence network map of the primary keyword "nanobubble" (page 5). Figure 3 shows country-level publication distribution (page 5). Figure 4 shows author-level publication distribution (page 6).	Pages 6-10
	13d	Narrative synthesis was used, following the SALSA approach (Scope, Appraisal, Literature Search, Analysis). This approach was chosen because included studies are heterogeneous in design, soil type, gas type, and outcome measurement, making quantitative pooling inappropriate. No meta-analysis was performed.	Pages 6-10
	13e	Not applicable	
	13f	Not applicable	
Reporting bias assessment	14	Not formally assessed.	
Certainty assessment	15	Not applicable	
**RESULTS**			
Study selection	16a	2188 total records identified across four databases (Web of Science, ScienceDirect, MDPI, Scopus). 13 non-English records were excluded. 2175 English-language records screened by title and abstract. Records on water treatment, industrial applications, or unrelated uses were excluded. 72 publications selected for full-text review and 59 included in the final synthesis.	Page 3&4
	16b	Studies excluded after screening were focused primarily on: water treatment or purification, industrial NB applications, non soil related agricultural responses, and non-peer-reviewed sources.	Page 3&4
Study characteristics	17	All included studies are cited in the body text with author names, year, and reported findings. Full bibliographic details, including the journal, volume, page numbers, and DOI, are provided in the reference list.	Page 6-11
Risk of bias in studies	18	Not formally assessed.	
Results of individual studies	19	Not applicable	
Results of syntheses	20a	Not applicable	
	20b	Not applicable	
	20c	Not applicable	
	20d	Not applicable	
Reporting biases	21	Not formally assessed.	
Certainty of evidence	22	Not applicable	
**DISCUSSION**			
Discussion	23a	The findings confirm that NBSW has the potential to produce variable physical, chemical, and biological soil responses depending on gas type, soil texture, and application duration, rather than acting through a single uniform mechanism. These results are consistent with existing evidence	Page 10

Section and Topic	Item #	Checklist item	Location where item is reported
		on NB stability, gas solubility, and porous media transport, and extend prior individual studies by integrating oxygenation, nutrient dynamics, and structural soil responses within a single framework. Overall, NBSW shows agricultural potential, but its effects are very context-dependent, requiring gas type and application strategy to be suitable for specific soil conditions.	
	23b	Limited soil texture range (only silty clay loam and sandy loam studied), limited field-scale data, inconsistent patterns of downward water movement across studies, and a concentrated evidence base relying on a small number of studies.	Page 9&10
	23c	This review did not apply a formal risk of bias assessment to individual studies	Page 9
	23d	Testing NB gas types across a wider range of soil textures and timescales; clarifying why NB-induced restriction of downward water and solute movement is consistent in some soils but not others; and connecting NB related soil changes more directly to plant physiological and yield outcomes.	Page 9
**OTHER INFORMATION**			
Registration and protocol	24a	This review was not registered in a public registry prior to data extraction.	
	24b	No protocol was prepared before conducting this review.	
	24c	Not applicable	
Support	25	No external funding	
Competing interests	26	The authors declare that they have no known competing financial or personal interests that could have influenced the work reported in this review.	
Availability of data, code and other materials	27	This review article is based on an analysis of the existing literature. No original data or models for this study were generated.	Page 11

From: Page MJ, McKenzie JE, Bossuyt PM, Boutron I, Hoffmann TC, Mulrow CD, et al. The PRISMA 2020 statement: an updated guideline for reporting systematic reviews. BMJ 2021;372:n71. doi: 10.1136/bmj.n71. This work is licensed under CC BY 4.0. To view a copy of this license, visit https://creativecommons.org/licenses/by/4.0/
